# Erratic Flu Vaccination Emerges from Short-Sighted Behavior in Contact Networks

**DOI:** 10.1371/journal.pcbi.1001062

**Published:** 2011-01-27

**Authors:** Daniel M. Cornforth, Timothy C. Reluga, Eunha Shim, Chris T. Bauch, Alison P. Galvani, Lauren Ancel Meyers

**Affiliations:** 1 Section of Integrative Biology, The University of Texas at Austin, Austin, Texas, United States of America; 2 Department of Mathematics, Pennsylvania State University, University Park, Pennsylvania, United States of America; 3 Department of Epidemiology and Public Health, Yale University School of Medicine, New Haven, Connecticut, United States of America; 4 Department of Mathematics and Statistics, University of Guelph, Guelph, Ontario, Canada; 5 Santa Fe Institute, Santa Fe, New Mexico, United States of America; Penn State University, United States of America

## Abstract

The effectiveness of seasonal influenza vaccination programs depends on individual-level compliance. Perceptions about risks associated with infection and vaccination can strongly influence vaccination decisions and thus the ultimate course of an epidemic. Here we investigate the interplay between contact patterns, influenza-related behavior, and disease dynamics by incorporating game theory into network models. When individuals make decisions based on past epidemics, we find that individuals with many contacts vaccinate, whereas individuals with few contacts do not. However, the threshold number of contacts above which to vaccinate is highly dependent on the overall network structure of the population and has the potential to oscillate more wildly than has been observed empirically. When we increase the number of prior seasons that individuals recall when making vaccination decisions, behavior and thus disease dynamics become less variable. For some networks, we also find that higher flu transmission rates may, counterintuitively, lead to lower (vaccine-mediated) disease prevalence. Our work demonstrates that rich and complex dynamics can result from the interaction between infectious diseases, human contact patterns, and behavior.

## Author Summary

When influenza spreads through a human population, its dynamics are shaped by both the complex patterns of contact that arise through our daily activities and individual decisions about the prevention and treatment of flu infections. However, until recently, mathematical models of flu transmission have ignored complex interaction and behavioral patterns in order to facilitate mathematical analyses. Here, we combine two recent approaches to modeling flu–network theory and game theory–to address the interplay between contact patterns and host vaccination decisions during seasonal flu outbreaks. Intuitively, the more contacts one has, the more likely he or she is to vaccinate. However, under the assumption that people make rational decisions based on complete information about the prior seasonal epidemic, vaccination decisions are predicted to vacillate dramatically. A severe epidemic in one year inspires high vaccination rates in the following year; this causes a milder epidemic which then leads to lower vaccination rates in the following year; and the cycle begins anew. We find further that the more homogeneous the contact patterns, the more pronounced the vacillations will be, and that decision-making based on multiple past seasons (rather than just one) leads to much more consistent behavior.

## Introduction

Human decision-making profoundly impacts the spread of disease as well as the effectiveness of intervention measures. However, decision-making has rarely been incorporated into mathematical modelling of epidemics [1], [2]. Vaccination not only protects vaccinated individuals but also breaks chains of transmission that would otherwise occur, thereby indirectly protecting individuals who have not been vaccinated. Perceived risks associated with disease and vaccination may critically influence adherence and thus the ultimate fate of an epidemic. Although federal mandates minimize these effects for some diseases, for recurring epidemics like seasonal influenza whose control relies on voluntary vaccination, our model predicts that there may be a mutual feedback between behavior and disease: high prevalence in one season may increase future vaccination, thereby lowering future prevalence; conversely low prevalence may decrease future vaccination, which ultimately increases prevalence.

Recently, methods from classical game theory have provided valuable insights into interactions between epidemiology and decision-making [3]. For example, the elderly have high risks of developing severe or fatal disease when infected by seasonal influenza and thus are expected to vaccinate at high rates; younger people, on the other hand, perceive lower risks and thus less incentive to vaccinate [4]. However, school-aged children have more social contacts than the elderly and can therefore transmit the disease in much higher numbers [5]. Game theoretical approaches have also shown that herd immunity, or the indirect protection of vaccination, can lead to a so-called “free-rider” problem. Individuals may forgo vaccination when they perceive that they are sufficiently protected by the immunity of others. This poses a problem for voluntary vaccination programs, because the best strategy for a community as a whole may be very different from that for short-term, individual self-interest. In particular, complete eradication by voluntary vaccination may be undermined by self-interested behavior [2]. However, this problem can be averted using a fast and reliable ring vaccination strategy that targets infected individuals and their contacts, as has been shown for smallpox [6]. This analysis shows that introducing contact network structure can significantly alter the predicted impact of rational behavior on vaccine coverage levels.

Here, we explore the interrelationship among network structure, vaccination decisions and annual influenza dynamics. Specifically, we investigate the impacts of (1) contact heterogeneity, (2) the transmission rate of flu and (3) the number of prior seasons considered (remembered) when individuals make vaccination decisions on the threshold number of contacts above which individuals are expected to vaccinate and the resulting fraction of the population expected to vaccinate. We have developed a mathematical framework for studying influenza vaccination strategies in a population with explicit network structure that experiences seasonal epidemics and use cobwebbing [7] to predict oscillatory patterns in vaccination behavior. We show that as the transmissibility of the disease increases, the equilibrium state evolves from a fixed strategy into a two-strategy oscillation and back into a fixed strategy. In reality, however, influenza vaccination patterns are relatively stable. We explore several explanations for the discrepancy between our model and the observed patterns and show that predicted oscillations dampen when individuals make vaccination decisions based on multiple prior years of experience.

## Methods

### Network Theory Background and Epidemiological Calculations

We model the interplay between the seasonal transmission of influenza and human vaccination behavior using percolation theory applied to contact networks. In general, network-based models have shown that contact patterns can dramatically impact disease dynamics [8], [9]. In a network model, nodes represent individuals; edges connecting nodes represent contacts that can lead to disease transmission; the number of edges coming out of a node is called its degree; and the distribution of these values is called the degree distribution. Whether an outbreak will grow into an epidemic depends on the degree distribution. All else equal, the greater the variance in contact rates, the more vulnerable the population [10]. Furthermore, an individual's risk of infection during an epidemic increases with the individual's degree [9]. Therefore, with perfect information, individuals should consider not only their own contact patterns but the local and overall structure of their community when they decide whether to vaccinate.

We build on the theory of epidemics on infinitely large random graphs [10], in which the fate of any outbreak is determined by the distribution of degrees within the network and the probabilities of transmission across the edges in the network. In a network where the degrees of individuals are independent of those of their contacts, the degree distribution can be represented by 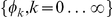, where 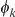 is the probability that an arbitrary individual from the population has 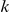 potential transmission contacts with other individuals. Additionally, each edge (from a node 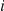 to a node 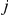) has a transmissibility, that is a probability that, if infected, 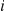 will transmit disease to 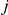 during its infectious period; here we assume individuals gain immunity upon recovery. If the transmissibilities can be assumed to be independently and identically distributed (i.i.d.) random variables on the edges of the network, then one can calculate the expected size of an epidemic and other key epidemiological quantities based solely on the expected (average) transmissibility 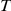 across edges in the network. This iid assumption breaks down if there is variation among nodes in terms of infectiousness and/or susceptibility, because their edges will have correlated probabilities of transmission [11], [12].


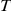
 summarizes several aspects of disease spread including the frequency of encounters between connected individuals, the length of infectious period, and the probability that a given encounter will lead to transmission. Here, we assume that the 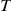 values are constant from year to year and thus ignore virus evolution as well as naturally acquired cross immunity. Our analyses focus primarily on the fairly wide range of transmissibility values that have been estimated for seasonal influenza (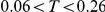) [13].

The size of an epidemic started on a random network depends in part on the transmissibility 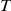. For a given degree distribution 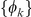, there is a critical transmissibility
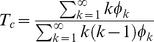
(1)below which a population is expected to experience only small outbreaks, and above which large epidemics are possible but not guaranteed. If 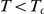, the number of infected individuals in the epidemic is finite with probability 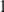. If 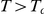, then there is a nonzero probability that a positive fraction of an infinitely large population will become infected. The derivation of 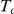 and other important epidemiological quantities in the network framework are given in [10] and [14]. Note, that this theory predicts behavior in a *typical* large random network with the specified degree distribution; and the predictions will be inexact for networks with extensive clustering or modularity.

### Network Degree Distributions

We compare vaccination dynamics across three different classes of networks: a pseudo-empirical network based on estimated contact patterns in an urban setting, a homogeneous network in which all individuals have almost identical degree, and a highly heterogeneous (scale free) network in which degrees follow a truncated power law distribution. Although flu networks are neither completely homogeneous nor scale free, these comparisons allow us to investigate the importance of network structure on the interaction between vaccination behavior and flu transmission dynamics. Our pseudo-empirical *urban network* is based on a simulation of urban contact patterns using empirical census, mobility, school, health care, employment, and other relevant data. This distribution has been used previously to study the spread of diseases through typical urban populations [9], [13], [15]. It is bimodal with adult and school-aged children having mean degree of approximately 20 and young children having a lower mean degree between 5 and 10. The homogeneous network was generated by applying a homogenizing procedure to the urban degree distribution. Specifically, given degree distribution 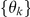, its 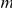
th homogenization (with 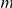 odd) has distribution 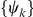, where
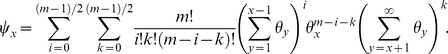
This is the expected distribution if one were to calculate the medians of 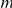 random variables drawn from the degree distribution 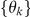 many times. The bigger the sample 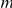, the closer the median of the sample is the to median of the baseline degree distribution; and thus as 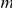 increases, the resulting homogenized distribution converges to a delta function on the integers. Our *homogeneous network* is the 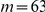 homogenization of the urban network degree distribution. Power law networks are characterized by a majority of low degree individuals and a minority of very highly connected individuals (having much higher degrees than found in more homogeneous networks). We chose the parameters of our *power law network* so that it has the same critical transmissibility (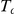) as the urban network (0.055) [9]. (The homogenized urban distribution network has a slightly higher critical transmissibility (0.062).) Specifically, the probability of degree 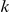 in the power law network is given by
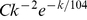
(2)truncated for 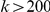, where 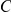 is a normalizing constant. All three distributions are shown in Figure 1B.

**Figure 1:**
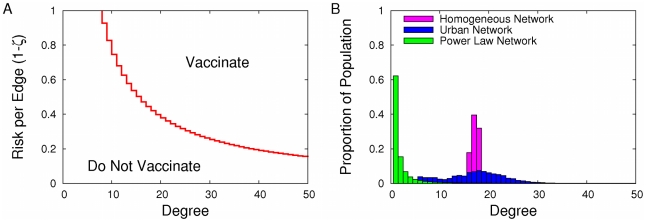
In a heterogeneous population, an individual's decision to vaccinate depends on the number of his or her contacts (degree) and the perceived epidemiological risk in the prior season. (A) When the costs of vaccination and infection are the same for everybody, an individual should only choose to vaccinate if his or her risk exceeds a calculated threshold depending on the person's degree (here 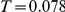). (B) The proportion of the population with each given degree are different for a homogeneous network (magenta histogram), an urban network (blue bimodal histogram), and an exponentially-scaled power-law network (steeply descending green histogram). For a log-linear plot of the degree distributions, see the online supplement.

### Vaccination Dynamics

To study the vaccination behavior of individuals, we adopt a behavior model similar to that of [16]–[18]. In our model, each individual decides to vaccinate based on knowing his or her degree and the average per-contact risk of infection in prior seasons; he or she does not directly consider personal infection history or the histories of immediate neighbors. Individuals weigh their perceived costs and benefits of vaccination and decide whether to vaccinate accordingly. Let 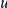 be the baseline component of the payoff corresponding to the value of a healthy life over the course of an epidemic.

An individual's cost of vaccinating (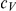) includes the monetary cost of vaccination, the perceived vaccine risks (reflecting both the perceived probability of adverse events and their perceived severity), long term health impacts, and other intangibles. Perceived vaccine risks need not reflect actual vaccine risks. The cost of infection (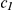) also has a monetary component corresponding to factors including missed work days, as well as the costs of doctors visits and medication. Influenza vaccines are only partially effective, that is, some vaccinated individuals remain susceptible to infection. We assume that, if infected, a vaccinated individual experiences less severe disease, but is equally infectious as a non-vaccinated, infected individual. We represent this in our model with a reduction in the severity (costs) of infection, 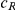. In our analysis, we assume perceived values (Table 1) that have been estimated from survey studies [4] and actual vaccine costs from [19]. Besides the costs of the infection and vaccination, individuals are also aware of their number of contacts (degree), and make independent estimates of the risks of each outcome based on their degree.

**Table 1:** Model parameters describing network structure and cost structure.

symbol	description	value
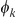	the probability that a random individual has degree 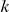	
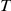	average probability that an infected contact infects an unvaccinated individual during his/her infectious period	
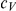	actual cost of vaccination	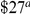
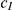	perceived cost of infection to an unvaccinated individual	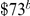
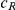	perceived cost of infection to a partially resistant (immune) person	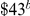
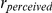	perceived efficacy – probability that vaccination yields immunity	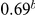
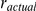	actual efficacy – probability that vaccination yields immunity	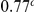

All must be specified a priori. ((a) Luce et al. [19], TIV costs Table 2, (b) Galvani et al. [4] (weighted by age in urban population), (c) Bansal et al. [13], Table 2 (weighted by age in urban population).)

The vaccination strategy 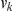 of an individual of degree 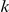 is the probability that the individual will be vaccinated. The payoff to an individual of degree 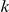 as a function of its vaccination strategy 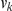 is given by the payoff function
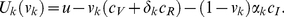
(3)where 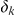 is the perceived probability of becoming infected if vaccinated and 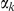 is the perceived probability becoming infected if not vaccinated. (We assume 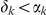). Here, we implicitly allow individuals to adopt mixed strategies such that 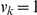 means always vaccinate, but 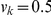 means that the individual chooses to vaccinate with probability 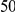
%, choosing randomly each time. We also assume that vaccination has no benefits beyond those related to the current epidemic period. Given Eq. (3), we can determine the strategies 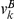 that maximize the payoff to an individual for given risks of infection in terms of the costs and risks (see online supplement). We use the term *rationality* to refer to our assumption that individuals make vaccination decisions that yield the highest personal utility (based on their perceived risks). A utility function could also include family health and a wealth of other factors, but, for simplicity, we have assumed that individuals behave according to self-interest.

To make use of these payoff rules, individuals must assess their risks of infection with (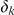) and without (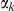) vaccination. Our model assumes that individuals estimate these risks based on past information and their own degree, which we assume individuals know accurately. In the simplest case, we assume that individuals use the observed distributions of influenza cases in the prior season to compute the probability that they will become infected in a future season. That is, they operate under the assumption that attack rates will be similar from one season to the next. These estimates will be incorrect to the extent that vaccination patterns differ from the prior season.

The estimation of epidemiological risks depends on a key variable: the probability that a random contact remains uninfected throughout an epidemic (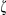). Suppose that a fraction 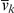 of individuals with degree 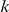 are vaccinated, and that a fraction 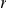 of vaccinated individuals acquire immunity to infection, whereas 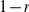 of vaccinated individuals remain fully susceptible. (Note that if instead we were to assume that vaccination imparts a certain degree of protection per contact, then high degree individuals might not have sufficient incentive to vaccinate with imperfect vaccines because they would likely maintain a high level of epidemiological risk. See Sec. 4 of online supplement for a brief discussion of this.) The probability of infection for an unvaccinated individual with degree 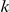 is given by
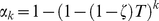
(4)
[8] and using the vaccine efficacy term 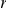 from above, the probability of infection for a vaccinated individual of degree 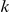 is
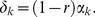
(5)To find the probability 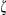 of a random contact avoiding infection, based on the epidemic in the prior season, we solve the following self-consistency equation (derived in Text S1)
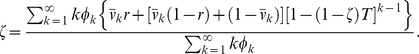
(6)where the vaccination fractions reflect behavior in the prior season.

Thus, 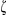 is the cornerstone of the individual risk assessment component of the decision process. Because 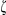 depends on the net vaccination rates of individuals of different degrees, the payoff function for individuals of degree 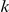 depends not only on the individual's current vaccination strategy 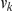, but also on the net vaccination rates for all degrees 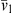, 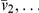. Thus, we rewrite the payoff functions as 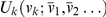. In trying to maximize their payoffs, individuals decide to vaccinate at the start of an influenza season when the benefit of vaccination in preventing infection exceeds its costs; and they base their estimates of current risk on the prevalence in the previous season. An individual can approximate 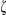 based on how many contacts were sick in the previous season, however we make the assumption here that individuals gauge 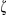 accurately.

Figure 1A illustrates that higher degree individuals perceive greater overall risk of infection and are thus more likely to vaccinate. (We believe that risk (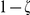), the probability that a random contact was infected during the prior season, is a more intuitive quantity than 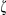 and use it in the diagrams throughout this paper.) The threshold between vaccinating and not vaccinating moves to lower and lower degree as the risk per edge increases. For example, at a perceived risk of 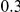, individuals with at least 25 contacts are expected to vaccinate, whereas at a perceived risk of 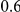, this threshold drops to a degree of 12.

We also consider an extended version of the model in which individuals infer their infection risk from several past epidemics rather than just from the prior season. Let 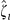 be the perceived probability of a random contact not becoming infected in year 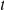, and 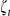 to be the actual probability of a random contact not becoming infected. A more general model for the dynamics of perception is then given by the recursive equation
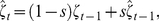
(7)where the parameter 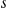 controls the duration of memory: 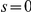 means that individuals base their behaviors on only the most recent epidemic's size (the simple model), 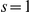 means individuals ignore the recent data in favour of their initial belief, and intermediate values mean that individuals weigh information across all prior epidemics, with an emphasis on more recent seasons. In both the simple and extended models, individuals are essentially making decisions based on infection risk in previous seasons. These and other model parameters and variables are summarized in Tables 1 and 2.

**Table 2:** Other variables used when describing the transmission network structure.

symbol	description
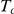	critical transmissibility threshold
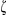	probability a random contact is not infected per epidemic
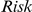	probability of a random contact being infected per epidemic (that is, 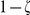 )
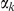	probability that an unvaccinated individual of degree 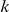 becomes infected
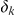	probability that a vaccinated individual of degree 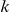 becomes infected
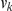	probability an individual of degree k will vaccinate

## Results

To examine the interplay among network structure, decision-making, and disease spread, we compare vaccination and disease dynamics of the above model in our urban and powerlaw networks. We start by assuming that individuals only use the previous season for assessing their risks (that is, risk is determined by Equation (7) with 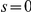). Figure 2 illustrates the basic dynamics of our model and the impact of network structure on them. Our calculations show that if individuals update their vaccination strategies each season in response to changes in their perceived risk, then the actual risk itself will change from one season to the next (Figure 2A). High risk in the prior season will lead to high vaccination rates and thus low disease risk in the current season, and vice versa. A strategy set 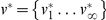 is a Nash equilibrium if individuals of each degree are using a best-response strategy and the annual risk 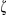 is not changing; rational responses to individual-level perceptions of risk lead to vaccination patterns that are constant from one epidemic period to the next. The Nash equilibrium always takes the form 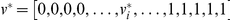 with 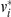 in 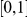 as shown in the online supplement. Figure 2B shows the impact of network structure (degree distribution) on Nash equilibria levels of risk and the threshold degrees above which everyone vaccinates at these equilibria. In this case (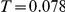), the Nash degree threshold for vaccination increases with the heterogeneity of the degree distribution; however the fraction of the population predicted to vaccinate at the Nash equilibrium does not change monotonically with network heterogeneity (6.8% vaccinate in the urban network, followed by 0.75% and 0.066% in the homogenized and power law networks, respectively).

**Figure 2:**
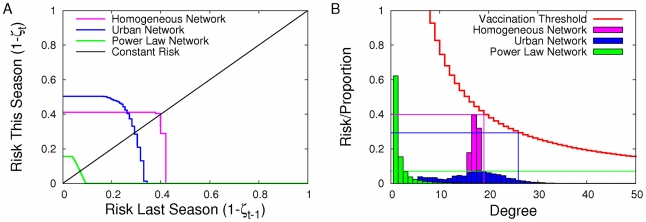
Risk from one season to the next and equilibria in the homogeneous, urban and power-law networks. (A) The inter-seasonal risk map showing the relationship between risk in one season and risk in the next season, assuming everybody acts to maximize his or her payoff (as in Figure 1A). The line 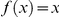 indicates constant level of risk from one season to the next, and an intersection of the response risk curve with this line represents a Nash Equilibrium. The stairstep shape seen in the homogeneous and urban networks is also present in the power-law example but appears smooth here as the steps are very small in comparison to the line width. (B) Each of these intersection points corresponds to an equilibrium level of risk (horizontal lines) and vaccination threshold degrees (vertical lines). Both figures assume that transmissibility is 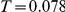.

Our urban network has most individuals with between 10 and 30 contacts, and thus with our model represents a network ranging between moderate and high levels of epidemiological risk. Here, at equilibrium, the degree threshold for vaccination is relatively low (about 26 contacts) a proportion of 0.07 of the population vaccinates (higher than in the power-law case), but those individuals not vaccinating are well enough connected to keep population-wide risk relatively high (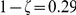). In contrast to the urban network, the power law network is comprised of a majority of low degree individuals with few opportunities for disease transmission and a small but important minority of very high degree individuals that can readily protect themselves through vaccination. Thus, relative to the urban network, the scale free (power law) network has a lower equilibrium level of risk (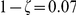), higher degree threshold for vaccination (about 109 contacts), and a smaller fraction of the population that is expected to vaccinate (0.0007). Our homogenized urban network has fewer high degree individuals than the urban network, so the population has lower incentive to vaccinate. At Nash equilibrium, the proportion vaccinating is lower (0.007) and the epidemiological risk is higher than in the urban network. Although this model always has a unique Nash equilibrium, the dynamics need not converge to this equilibrium, as described below.

When we take a closer look at these dynamics in the more realistic urban network across three different levels of transmissibility (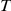), we find that when risk in the prior season is lowest (zero), individuals perceive no risk and thus do not vaccinate for the current season (Figure 3). Consequently, disease sweeps through the population unhindered by vaccination, resulting in the maximum possible transmission (risk). Although the equilibrium risk level is not generally monotone in the transmissibility (Figures 3A, 3B), the equilibrium level of vaccination does monotonically increase with transmissibility (Figure 3D), which will always be the case for the given Eq. (4) and (5) (see Text S1). Changes in transmissibility affect risk and ultimately the Nash equilibria vaccination thresholds (Figure 3B). The equilibrium risk at very low and very high transmissibilities is similar, indicating that, in terms of risk, additional vaccinations can compensate for the increased transmissibility and 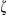 (Figure 3C).

**Figure 3:**
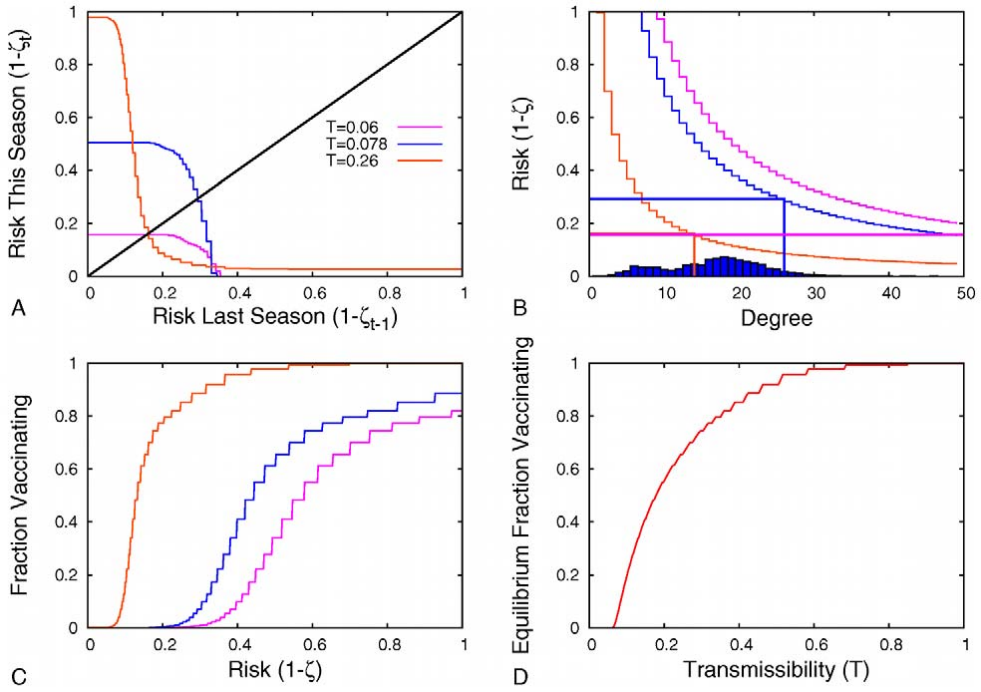
The effect of transmissibility on risk and vaccination dynamics. (A) The effect of transmissibility (T) on inter-seasonal change in risk in the urban network. Equilibria occur at intersections with black (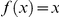) line. (B) As the transmissibility increases, the equilibrium vaccination threshold and risk change non-monotonically. For low T (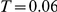, magenta) the equilibrium level of risk is less than 0.2 per year, and the vaccination threshold is greater than the maximum degree in the network. Consequently, nobody in the population is expected to vaccinate. For intermediate T (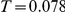, blue), the equilibrium risk is near 0.5 per year and only a small fraction of the most connected individuals vaccinate. At high T (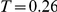, orange), a large fraction of individuals vaccinate, leaving an intermediate level of risk. (C) As both risk and transmissibility increase, vaccination behavior increases. (D) Consequently, the equilibrium level of vaccination is an increasing function of the transmission rate.

Figure 4A shows a cobwebbing diagram to illustrate the dynamics in a particular example and how convergent and dynamic stability can be determined from it and similar plots in Figures 2A and 3A. Dynamic stability refers to whether or not a very slight perturbation will cause oscillations in a population at equilibrium. In some cases, dynamic instability corresponds to a situation where the equilibrium requires a degree class to be partially vaccinating. As individuals base their decisions on 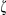 and their degree, there can only be one partially vaccinating degree class: individuals with higher degree will get vaccinated, while those with lower degree will not. Within the partially-vaccinating degree class, the payoff if vaccinating must be identical to the payoff if not vaccinating because Eq. (3) is linear in 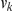. However, any arbitrarily small perturbation in risk is sufficient to make the degree class either fully vaccinate or not vaccinate at all. When there is no partially vaccinating class, that is, when the threshold cleanly divides the population into two groups, arbitrarily small perturbations to risk have no such effect. Convergent stability is important also, as it indicates whether populations close to the Nash equilibrium evolve toward it. If the equilibrium is convergently unstable but there is no partially vaccinating degree class, a population sitting on the Nash equilibrium will stay there due to the disincentive for anyone to change their strategy. However, any small forced perturbation away from the Nash equilibrium (such as that caused by environmental stochasticity) will cause the population to move away from the Nash equilibrium. If the population is convergently stable, but the Nash equilibrium has a partially vaccinating degree class, we expect the population to move toward the equilibrium and end in reaching a 2-cycle with only one degree class switching between vaccination and non-vaccination and other classes maintaining constant behaviors.

**Figure 4:**
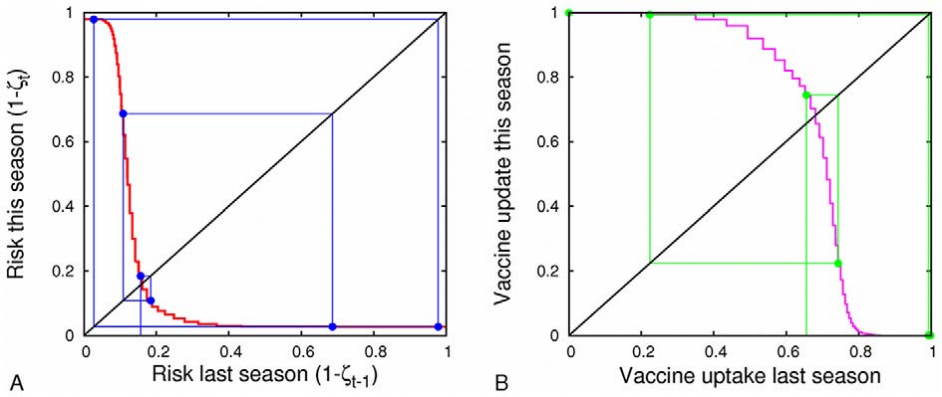
Cobwebbing diagrams of risk and vaccination rates. (A) shows the inter-seasonal risk relation and (B) the corresponding fractions of the population expected to vaccinate as a best response to the perceived risk of infection for the urban network when 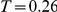 and 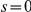. Whether vaccination rate (or 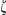) is stable from season to season depends on the slope of the inter-seasonal risk relation at the equilibrium (the slope of the intersections above or likewise in Figures 2A and 3A. When this slope is zero, there is a partially vaccinating degree class at equilibrium and the system is dynamically unstable, and otherwise (infinite slope) there are no partially vaccinating classes and it is dynamically stable. Additionally, when the “average” slope has magnitude less than one, the system is convergently stable. Conversely, if the magnitude of the average slope is greater than one, it is not convergently stable. The dynamics shown are both dynamically and convergently unstable.

Steady-state risks (corresponding to Nash Equilibria) and unstable limit cycles change as transmissibility increases for our homogenized network, simulated urban network, and power law network (Figure 5). Although we focus our discussion on the dynamics of risk, we also plot prevalence (proportion of population infected) (Figure 6). For intermediate transmissibility values, risk is predicted to oscillate considerably from one season to the next, with the lower branch reaching close to zero (Figure 5). As noted above, this indicates virtually no vaccination in one season followed by wide-spread disease and consequently high vaccination in the next. The dynamic behavior over the whole transmissibility interval differs significantly from the bifurcations seen by [7]. Rather than exhibiting the common period-doubling cascade to chaos, the bifurcation pattern exhibits a period-bubbling pattern [20] that returns to steady-state for large transmissibilities. In fact, it is not possible to achieve an orbit of more than two oscillation points in our model when only one season is considered (see Text S1). For transmissibilities below the critical transmissibility value (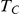) of 0.055, there are no epidemics, and so risk is always zero. After this cutoff, the Nash risk rises quickly as epidemics then become possible. At this point we see a branching of orbit values, where the Nash solution is no longer dynamically stable. The curve representing Nash solutions takes on a sawtooth pattern. Higher transmissibilities yield higher vaccination rates at the Nash equilibrium (see Text S1). The decreases of the Nash curves in Figure 5 are caused by increasing vaccination rates of partially-vaccinating degree classes. Recall that the Nash solutions often contain one partially vaccinating degree class; the increase of vaccinators within a given class more than compensates for the increase in transmissibility, causing a decrease in risk. Increases in the diagrams correspond to cases when the changes in strategies are not sufficient to counterbalance the increases in transmissibility or when increases in transmissibility have no effect on the vaccination strategies at equilibrium. There are two causes for this second situation: newly vaccinating degree classes may contain no individuals and thus have no effect on risk; and some values of transmissibility are between the point that a degree class 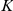 is fully vaccinated and that at which 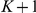 would begin vaccinating; that is to say, there are no partial vaccinating classes.

**Figure 5:**
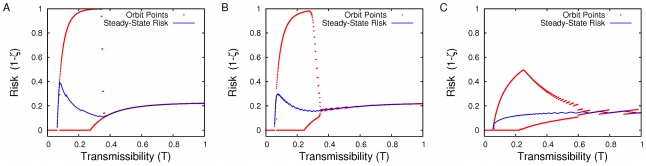
The effect of transmissibility on risk. The impact of transmissibility on risk (steady-states and orbits) for the (A) homogeneous (B) urban and (C) power-law networks. Nash equilibria are unstable for the majority of the interval between 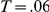 and 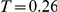; some individuals waver between accepting and rejecting vaccination.

**Figure 6:**
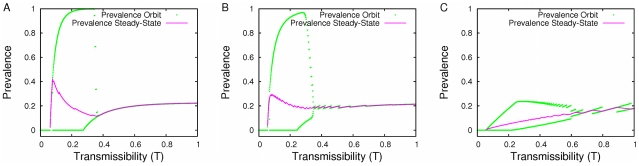
The effect of transmissibility on prevalence. The impact of transmissibility on prevalence (steady-states and orbits) for the (A) homogeneous (B) urban and (C) power-law networks.

We also found that the oscillations in the homogeneous and urban networks are more extreme at low 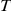 values than in the power law network, yet they stabilize at high 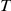 values. The steady-state values also show different trends: they exhibit a sharp decrease around 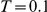 in the urban network and an even sharper decrease in the homogeneous network, but generally increase with 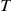 in the powerlaw network. Figure 6 shows the same pattern in prevalence: the increase of prevalence with transmissibility in the power law network and the surprising *decreases* of prevalence in the homogeneous and urban networks. These differences stem from the larger number of medium degree individuals in the homogeneous and urban network compared to the power law network. At low values of 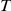, vaccination and behavioral fluctuations occur only in high degree classes. Although the power-law network has more extremely high degree individuals, it has very few of them, and they vaccinate almost immediately when 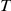 is increased; thus compared to the more homogeneous networks, it experiences less extreme epidemiological oscillations. At higher values of 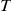, moderate to high degree individuals vaccinate and behavioral fluctuations occur in relatively low degree classes. As low degree groups are much more numerous in the power-law network than in the other networks and low degree individuals have very few contacts to whom they can transmit disease, orbit points converge to the Nash strategy in the homogeneous and urban networks, whereas the power law network continues to experience oscillations even at very high 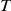
's because they have many more low degree individuals. While the homogeneous and urban networks exhibit similar dynamics, there are a few differences. In the homogeneous network, equilibrium risk and prevalence reach higher levels (both near 0.4). Compared to the urban network, relatively few of the individuals in the homogeneous network have incentive to vaccinate. The steady-state lines are also less jagged in the homogeneous network because there are fewer degree classes whose behavior can change as transmissibility increases.

So far, we have assumed that individuals predict current risk based solely on prevalence during the prior season. While this provides valuable insights into the potential impacts of past behavior and disease spread on future decisions and transmission, it is unlikely that people base their decisions on such simple considerations. Thus we also consider cases of Eq. (7) where 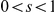, corresponding to a geometric-discounting of historical epidemic risks. Recent seasons are thereby weighted more heavily than more distant seasons, with 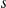 controlling the historical inertia (Figure 7A). When we extend the time-horizon for decision making in this way, the oscillations begin to disappear (Figure 7B). In Figure 8, we see the effect of varying 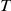 for different 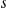 values. The general pattern is that the larger 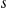 is, the less inter-seasonal variation there is, and in fact it converges upon the steady-state strategy. Additionally, the larger the value of s, the longer it takes the system to forget the initial conditions of our simulation; in the real world this might mean longer to forget an uncharacteristically high or low disease prevalence season.

**Figure 7:**
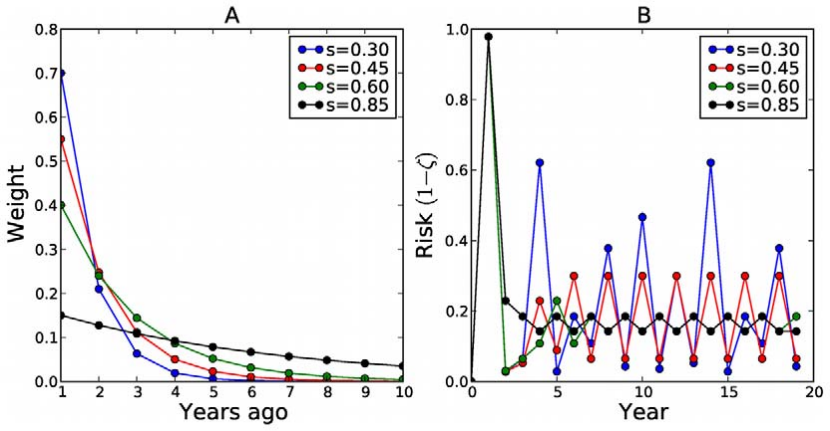
The effect of memory on vaccination decisions. (A) Contributions of past seasons to current perceptions of risk, under different s values. (B) The impact of prior information on oscillations when 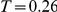: longer memory decreases variability.

**Figure 8:**
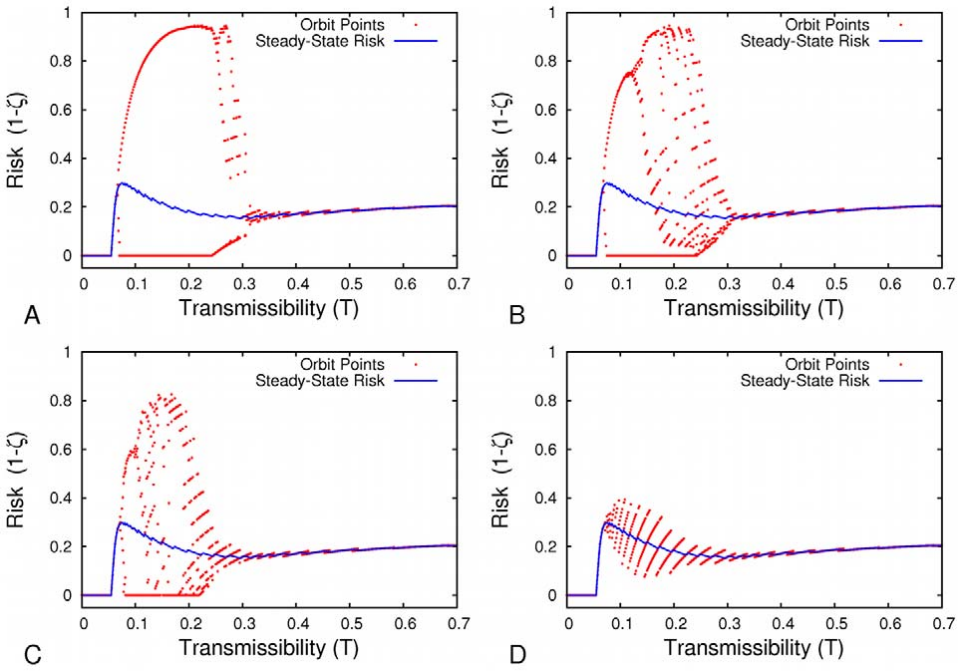
Longer-term memory reduces oscillations for various transmissibilities. As 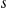 increases, individuals integrate more of their prior epidemiological experiences into their decision-making and two-cycles disappear. (A) 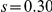 (B) 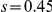 (C) 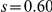 (D) 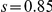.

Our results illustrate that network structure greatly impacts vaccine and disease dynamics, both in terms of the points of oscillation and the Nash strategy. They also show that, as individuals increasingly look backwards in time, these oscillations collapse onto the Nash strategy for all transmissibilities.

## Discussion

Network structure can have important effects on the spread of infectious diseases like influenza. Typically, high degree individuals are more quickly infected [21], but whether an individual becomes infected during an influenza epidemic depends not only on his or her contact patterns, but on the overall connectivity of the population [9]. Our model suggests that vaccination behavior similarly depends on both local and global connectivity. We have assumed that individuals have accurate information about the risk in the previous season and their own degree. In reality, individuals may approximate their risk by knowing how many of their contacts were previously sick or also by media sources, but, in our analysis, they know their own degree and the per-contact risk in the population in the previous season. Our comparison of a semi-empirical urban network and an exponentially-scaled power law network shows that, under the simplifying assumptions of our model, equilibrium behavior may be very different for individuals with identical numbers of contacts in the two networks. In the scaled power-law network, most individuals have very low degree and are unlikely to become infected. Thus even high degree individuals will perceive a relatively low risk, because few of their (low degree) contacts will have been infected in prior seasons. In the urban network model, contact patterns are more homogeneous and thus epidemiological risk is also more homogeneous.

In all cases, we find the likelihood that one vaccinate increases with degree, because the probability of infection increases with number of contacts (specified in Eq. (4) and Eq. (5)). This is consistent with observed correlations in vaccination behavior from one year to the next [22]. However, given that children tend to have higher numbers of contacts than adults, it is not necessarily consistent with the empirical observation that older adults vaccinate at the highest rates, followed by adults and then children [4]. While this discrepancy may reflect non-rational or public policy-driven behavior, it also stems, in part, from our simplifying assumption that risks associated with flu are homogeneous throughout the population. In fact, the severity of seasonal flu is thought to be highest for the youngest and oldest age groups [23]. This model can be extended easily to explicitly consider this pattern and other age-specific behavior, risks and perceptions; and such extensions may reveal complex interactions between sociological (network) and biological driven risks factors.

Our model also suggests that vaccination behavior may depend critically on the transmissibility of the circulating strain of influenza. For low levels of transmissibility, below the epidemic threshold, there is no epidemiological risk and thus nobody vaccinates. Just above the epidemic threshold, vaccination levels are predicted to converge to a stable Nash equilibrium, and the proportion of the population vaccinating increases as the transmissibility of the strain increases (Figure 3D). Individuals with more than a threshold number of contacts are expected to vaccinate, while those with fewer contacts are not. However, above a critical value of transmissibility, the model shows oscillatory vaccination behavior rather than an attracting equilibrium strategy. The population alternates between near-universal vaccination of all but the least connected individuals and vaccination limited to only the most highly connected individuals (Figure 6). Ultimately, at unrealistically high levels of transmissibility, the dynamics stabilize on a single equilibrium strategy. Differences in network shapes largely affect these characteristics; for example, in the urban network, increases in transmissibility may actually decrease prevalence, a trend not possible in our power law network (Figure 6) and one that seems not to be highlighted in previous models.

A natural question that emerges is whether a corresponding homogeneous-mixing model would exhibit these types of oscillations. Regardless of presence or absence of network structure, there may be a general propensity for such behavior-prevalence systems to oscillate, due to overcompensation mechanisms similar to those that cause oscillations in simple predator-prey models such as the Lotka-Volterra model. For instance, when disease prevalence is very low due to high vaccine coverage, rational individuals will stop vaccinating. The susceptible pool then grows and an outbreak occurs. Individuals begin to vaccinate again in large numbers, which thus drives the prevalence down to levels even lower than would occur if vaccine coverage were constant over time, and the cycle repeats. In empirically plausible parameter regimes of previous (non-network) behavior-prevalence models, little or no oscillatory behavior has been observed [4], [24]. However, in other parameter regimes, these and other non-network models oscillate in ways that mirror the oscillations observed in the present network model [24], [25]. When we compared our homogenized urban network with our original urban network, there were no major differences, but whether network structure per se enhances or suppress oscillations in behavior-prevalence systems is not yet clear and would be an interesting topic for future research.

To what degree are oscillations seen in reality? Annual rates are not constant, but neither are they oscillatory to the extent possible in the model [26]–[28]. There was rapid expansion in coverage in the 1980's and 1990's as seasonal influenza vaccines gained acceptance, followed by a saturation to approximately constant coverage [26], [28]. An autocorrelation analysis of these data sets indicates no statistically significant correlation at lag two, or any other lag up to and including five years. A straightforward, but unlikely explanation is that the transmissibility of influenza is below the level at which vaccination behavior is predicted to fluctuate. Estimates for the transmissibility of influenza approximately span the range 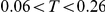 [13]. The explanation more likely rests on the assumptions of our vaccination decision model. In our initial model individuals make choices based solely on attack rates in the prior seasons. However, people use more heterogeneous decision heuristics and do not base their decisions exclusively on such information. Our model assumes that by knowing the number of their own contacts and per-contact risk in the population, individuals accurately estimate their own risks of infection in a comparable outbreak (same pathogen, same population, same levels of vaccination). In reality, one might not be able to estimate previous risk accurately and one may not properly assess his or her own likelihood of becoming sick even given perfect information about prior prevalence. Additionally, we assumed that individuals slightly over a particular risk cut-off vaccinate and those slightly under this cut-off do not. By contrast, the decision processes might be less sensitive to such small differences and possibly less deterministic. Lastly, some people might think back further in predicting their risk for the current season, and this degree of heterogeneity of memories may prevent oscillations.

In a study of measles vaccination, Philipson concludes that disease prevalence is an important factor in determining individuals' vaccination decisions [29]. Although our model assumes that this is also the case for influenza, there are likely many constraints on vaccine coverage other than individual choice, including vaccination policies, accessibility of immunization services, and having sufficient vaccine production capability to meet demand [30]. Individual choice itself likely depends also upon factors other than the attack rate in the previous season, such as the recommendations of physicians, the opinions of peers, an individual's state of health, and vaccine cost [27],[31]. Any tendency toward periodic cycles in vaccine coverage in the empirical data could easily be masked by these other determinants of vaccine coverage in real settings. Additionally, if behavior were modelled stochastically, so that greater marginal utility led to increased likelihood of vaccination, it would also likely dampen oscillation. Indeed, multi-season memory alone is sufficient to dampen the two-cycles observed in the model (Figure 8). The simplicity of our model allows a large degree of analytical rigour and cobwebbing facilitates comparison with classical models of population biology. Moreover, it demonstrates that global network structure can strongly influence not only the disease dynamics directly, but less directly as well by altering vaccination behavior.

## Supporting Information

Text S1 Derivations and theory.
(0.30 MB PDF)
