# Supplementary material for: Erratic Flu Vaccination Emerges from Short-Sighted Behavior in Contact Networks
Source: PLoS Comput Biol. 2011 Jan 27;7(1):e1001062. doi: 10.1371/journal.pcbi.1001062 (PMC3029241; doi:10.1371/journal.pcbi.1001062)
Supplement: Text S1 — Derivations and theory. (0.30 MB PDF) [file pcbi.1001062.s001.pdf]

1 Derivation of the probability of infection of a random contact

Here we explain the self-consistency formula Eq. (6). Recall that ϕ_k denotes the frequency of individuals with degree k and $\overline{v}_k$ denotes the fraction of degree k individuals opting to vaccinate. Suppose we start at a randomly chosen individual in a network and follow an arbitrary edge from that individual to a neighbouring contact. We wish to determine the probability ζ that this neighbouring contact is not infected. First, the relative likelihood of reaching a neighbour of given degree should be the frequency of that degree weighted by the number of contacts arriving at the vertex, so the probability that the neighbouring contact has degree k is $\frac{k\phi_k}{\sum_{k=1}^{\infty} k\phi_k}$. Suppose the neighbouring contact was not vaccinated. Given that $1-\zeta$ is the probability that an arbitrarily chosen susceptible contact was infected over the course of an epidemic and T is the probability that this infection would have been transmitted to the focal individual, then $(1-\zeta)T$ is the probability that the neighbouring contact transmitted infection to the focal individual. Extending this to the neighbours of the neighbouring contact, $(1-(1-\zeta)T)^{k-1}$ is the probability that a neighbouring contact of degree k did not become infected from one of its other contacts (other than the focal individual). So $(1-\overline{v}_k)(1-(1-\zeta)T)^{k-1}$ is the probability that an arbitrary neighbour of degree k was not vaccinated but did not become infected. On the other hand, if the neighbouring contact was vaccinated, then, with probability r, the contact has protective immunity and, with probability $1-r$, the contact remains susceptible after vaccination. If the neighbour remains susceptible after vaccination, then they avoid infection from one of their other contacts with probability $(1-(1-\zeta)T)^{k-1}$. So the probability that a vaccinated

contact of degree k does not become infected is

$$\overline{v}_k r + \overline{v}_k (1-r)(1-(1-\zeta)T)^{k-1}.$$

Combining all these terms, we find that ζ must satisfy the infinite-order polynomial equation

$$\zeta = \frac{\sum_{k=1}^{\infty} k\phi_k \left\{ \overline{v}_k r + [\overline{v}_k(1-r) + (1-\overline{v}_k)][1-(1-\zeta)T]^{k-1} \right\}}{\sum_{k=1}^{\infty} k\phi_k}$$

Alternatively, using generating function methods, one can derive ζ as the probability that a node at the end of a random edge in a Bethe lattice with given degree distribution and connectivity is a member of a finite cluster.

2 Proof that there are no p-cycles for $p > 2$ when $s = 0$

Theorem 1. *If decisions are only made based on previous season ($s = 0$), it is impossible to have an oscillation that is a p-cycle for any $p > 2$, regardless of the transmissibility T.*

Proof. Let A be the Nash Equilibrium. Now suppose we have x_1, x_2 be the first two ζ's in a cycle. Suppose $x_1 < A$. We note that this means $x_2 > A$ since the response of x_1 will have more vaccinators than A since the lower the initial ζ is, the greater the response ζ must be. As a result, subsequent x's must be going ever-closer to A or every-farther, and cannot be finite in number. So limit cycles must have one element (if it is A) or two elements. □

We note that this statement relies on the fact that higher ζ's lead to lower ζ's in response which is true when vaccines lead to a fractional decrease in infection probability, but not when the vaccine protection is a per-contact reduction as described below in Section 4. This does not mean the limit cycles are unique. There may be three or more period-2 cycles for

given parameter values. The topology implies that the number of cycles is always odd, with every pair of neighbouring stable cycles separated by an unstable cycle. Related results are found in [1].

3 Game Theory of Vaccination on the Simple Network Model

Given the main paper Eqs. (3), (4), and (6) for the payoff function, α_k, δ_k, and ζ, respectively, then a degree k individual's best-response strategy (that which maximizes their payoff for a given risk) is

$$\begin{aligned} v_k^B &= H(\alpha_k c_I - \delta_k c_R - c_V), \\ &= H([c_I - (1-r)c_R]\left[1 - (1-(1-\zeta)T)^k\right] - c_V), \end{aligned} \tag{S1}$$

where $H()$ is the Heaviside function

$$H(x) = \begin{cases} 0 & \text{if } x < 0, \\ [0,1] & \text{if } x = 0, \\ 1 & \text{if } x > 0. \end{cases} \tag{S2}$$

Theorem 2. *The random network epidemic model with vaccine efficacy independent of contact degree, $c_I > 0$, $c_R > 0$, and $c_V > 0$ has a unique Nash equilibrium.*

Proof. Let $\eta = 1-\zeta$ represent the probability of a random contacts infection if not vaccinated, a.k.a. the force of infection. A strategy set is a Nash equilibrium strategy set if and only if

it satisfies the vector-valued fixed-point inclusion relation

$$v^* \in v^B(v^*),$$

where

$$v_k^B(v^*) = H(\alpha_k(\eta(v^*), T)(c_I - (1-r)c_R) - c_V).$$

The general existence of a solution to this equation can be established using the Ky Fan inequality or Kakutani fixed point theorem [2]. Note that rather than having an arbitrary vector-valued relation (since $v^* = \{v_k^*\}$ is a vector), the special form of $\alpha(\overline{v}, T)$ and $\delta(\overline{v}, T)$ given by Eqs. (4) and (5) allows us to treat the best response function v^B as a function of the scalar probability $\eta(\overline{v})$ for random graphs; $v^* \in v^B(\eta(v^*))$. If we apply formulas for the probability of escaping infection $\eta(\overline{v})$ to both sides, we get the *scalar* inclusion relation $\eta(v^*) \in \eta(v^B(\eta(v^*)))$. Substituting $e = \eta(v^*)$, $e \in \eta(v^B(e))$ and equivalently $0 \in \eta(v^B(e)) - e$.

We can show that there is a unique solution e^* to this inclusion relation. First, we observe that the correspondence $\eta(v^B(e))$ is continuous. η is non-negative so if $e = 0$, $\eta(v^B(0)) - 0 \geq 0$. η is a probability less than 1, so if $e = 1$, $\eta(v^B(1)) - 1 \leq 0$. Suppose I_1 and I_2 are two subsets of the real numbers. We say $I_1 \leq I_2$ if and only if, for every $x_i \in I_1$ and $y_j \in I_2$, $x_i \leq y_j$. Now, the best-response vaccination probabilities are weakly increasing as the risk of infection increases in the sense that for each component k, $e_1 < e_2$ implies $v_k^B(e_1) \leq v_k^B(e_2)$. We also know that the less vaccination there is, the greater the risk of infection, so $e_1 < e_2$ implies $\eta(v^B(e_1)) \geq \eta(v^B(e_2))$ with equality holding only when $v^B(e_1) = v^B(e_2)$. Subtracting, we conclude that $\eta(v^B(e)) - e$ is strictly decreasing in e. These conditions imply that there is exactly one solution $e^* \in [0, 1]$ such that

$$0 \in \eta(v^B(e^*)) - e^*. \tag{S3}$$

Thus far, we have derived a necessary condition for a Nash equilibrium: if v^* is a Nash equilibrium, then $\eta(v^*) = e^*$. Obviously, there are many combinations of vaccination strategies that can result in an overall risk e^*. However, a Nash equilibrium must also be a best response, and the set of best responses (a subset of $[0,1]^{\mathbb{N}}$) is a continuous well-ordered set under the component-wise ordering relation. Specifically, if e_1 and e_2 are forces of infection with $e_1 < e_2$, then for $w_1 \in v_k^B(e_1)$ and $w_2 \in v_k^B(e_2)$, $w_{1,k} \leq w_{2,k}$ for all k but $w_1 \neq w_2$. This is ordering relies on α_k being strictly decreasing.

We also know that $\eta(v^B)$ is continuous, e^* is in the range of η, and η is strictly decreasing in v^B. If the set $v^B(e^*)$ has one point, that point is the unique Nash equilibrium. If $v^B(e^*)$ is not a single point, than it is a simply connected one-dimensional interval set. If $v^B(e^*)$ is a simply connected one-dimensional interval set, then the ordering of v^B and the monotonicity of η imply only one point in $v^B(e^*)$ can solve Eq. (S3). We conclude that there is always a unique Nash equilibrium. □

The strategy set of the Nash equilibrium varies, depending on the various parameters in the model. For instance, we can show that increasing transmissibility always increases the proportion vaccinating at equilibrium.

Theorem 3. *Suppose $v^*(T)$ is the Nash equilibrium strategy set as a function of the transmissibility T. If best responses are given by Eq.* (S1)*, then for every degree class k, $T_1 < T_2$ implies $v_k^*(T_1) \leq v_k^*(T_2)$.*

Proof. We know $\eta(v^*(T_1), T_1) \leq \eta(v^*(T_1), T_2)$ where $\eta(v^*(T_1), T_2)$ is the resulting risk of the strategy set $v^*(T_1)$ in a network with edge transmissibility of T_2 for instance. The related fixed-point map $f(x,T) = x - \eta(v^B(x,T),T)$ is monotonic increasing in x. Since

$\eta(v^*(T_1), T_1) \leq \eta(v^*(T_1), T_2)$, we also know

$$v_k^B(\eta(v^*(T_1), T_2), T_1) \geq v_k^B(\eta(v^*(T_1), T_1), T_1) = v_k^*(T_1)$$

for all k because the best response is increasing in risk for fixed transmissibility. Further,

$$v_k^B(\eta(v^*(T_1), T_2), T_2) \geq v_k^B(\eta(v^*(T_1), T_2), T_1) \geq v_k^*(T_1)$$

for all k since for a fixed risk, the best response is increasing in T. So

$$\eta(v^B(\eta(v^*(T_1), T_2), T_2), T_2) \leq \eta(v^*(T_1), T_2).$$

Rearranging, $\eta(v^*(T_1), T_2) - \eta(v^B(\eta(v^*(T_1), T_2), T_2), T_2) \geq 0$ Thus, $f(\eta(v^*(T_1), T_2), T_2) \geq 0$. Since for transmissibility T_2, $f(\eta(v^*(T_1), T_2), T_2) \geq 0$ and $f(\eta(v^*(T_2), T_2), T_2) = 0$, by monotonicity $\eta(v^*(T_1), T_2) \geq \eta(v^*(T_2), T_2)$ and thus $v_k^*(T_1) \leq v_k^*(T_2)$.

□

4 Alternative vaccine efficacy model

An alternative to the vaccine efficacy hypothesis studied in the main test is that vaccination or other prophylactic behaviour reduces the probability of infection *per contact.* For instance, a marginal antibody titer may protect against a small infectious dose of virus, but not a large infectious dose. If the probability that a random contact infects you is $(1 - \zeta)T$, then the probability of infection after vaccination is $(1 - \zeta)(1 - r)T$ where r is reduction in the probability of transmission. The probability of infection for an unvaccinated individual is given by

$$\alpha_k = 1 - (1 - (1 - \zeta)T)^k$$

and using the vaccine efficacy term r from above, the infection probability for a vaccinated individual is

$$\delta_k = 1 - [1 - (1-r)(1-\zeta)T]^k.$$

Given r and T and assuming that a proportion $zeta$ of contacts are not infected, the critical cost of vaccination above which individuals with degree k will not vaccinate is given by

$$c_V^*(k,\zeta) = [1 - (1-(1-\zeta)T)^k]c_I - \{1 - [1-(1-r)(1-\zeta)T]^k\}c_R. \tag{S4}$$

For small k,

$$c_V^* \approx c_I k \ln\left[\frac{(1-(1-r)(1-\zeta)T)^{c_R/c_I}}{1-(1-\zeta)T}\right], \tag{S5}$$

so c_V^* is increasing in k. For $0 < \{(1-\zeta)T, c_R/c_I, r\} < 1$,

$$\lim_{k\to\infty} c_V^* = c_I - c_R,$$

and the limit is approached from above with the vaccine efficacy r controlling the rate of convergence to the asymptotic critical vaccine cost. Thus, c_V^* has a maximum value as a function of k so long as the vaccine provides partial protection ($r > 0$) on a per-contact basis. Then if $c_V \in (c_I - c_R, \max_k c_V^*(k,\zeta))$, there is a range of intermediate degree classes for which vaccinations are the preferred behaviour (Figure S1). Individuals with many contacts are likely to be infected even after vaccination, and thus do not opt to vaccinate; and individuals with few contacts are unlikely to be infected even without vaccination, and thus do not opt to vaccinate. Thus, only individuals with intermediate numbers of contacts would have sufficient incentive to vaccinate. The inter-seasonal risk maps (Figure S2) lead to dynamics roughly similar to those observed under our original vaccine efficacy model (Figure S3).

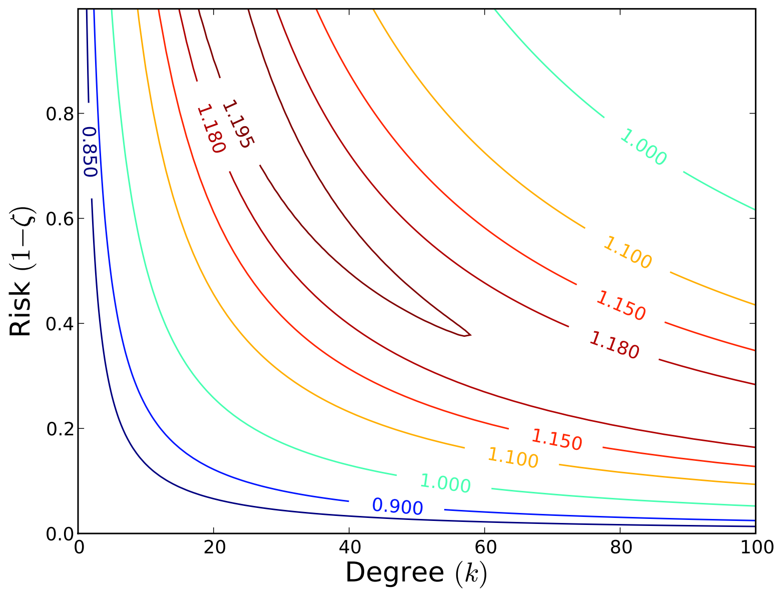

Figure S1: Vaccination windows for various vaccination costs c_V when vaccinations protect per contact instead of being degree independent for transmissibility $T = .078$. Vaccinating degrees are those between two lines of the same colour. and $1 - \zeta = 0.6$, only individuals with degrees between about 25 and 50 will opt to vaccinate.

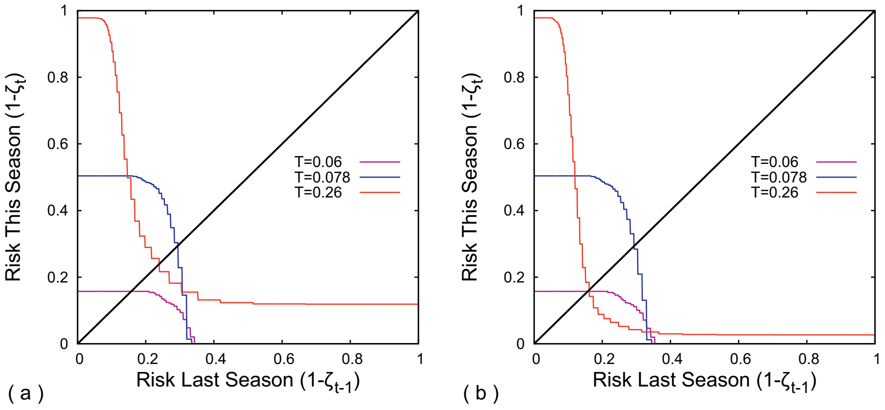

Figure S2: Comparison of inter-seasonal risk mappings under (a) the per-contact vaccine efficacy model and (b) the original all-or-nothing vaccine efficacy model, with all parameters equal. The plots appear similar, with the most notable difference being a thicker tail in the $T = .26$ case. Note, plot (b) is identical to Figure 3A in the main document.

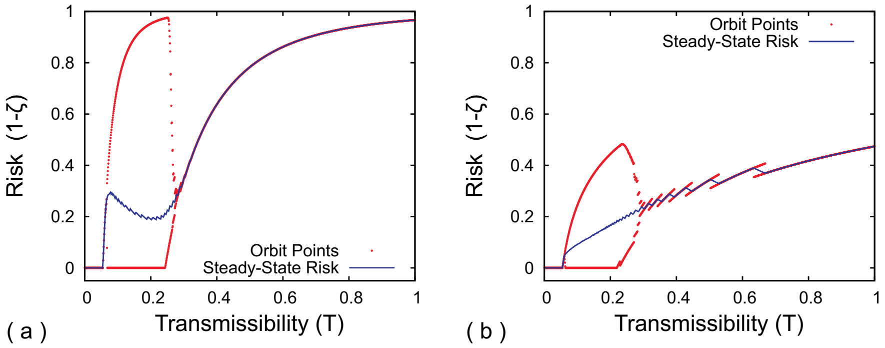

Figure S3: Bifurcation diagrams under the per-contact vaccine efficacy model for the (a) urban network and (b) scaled power law network. The power law network reaches equilibrium at slightly higher values of T than the urban network.

This window-effect breaks the conditions used to prove uniqueness of the Nash equilibrium above; and we conjecture that there may be multiple Nash equilibria in some scenarios. This also means that best responses are not generally monotone functions of the risk ζ, which also violates Theorem 3 (Nash equilibrium vaccination increases with transmissibility). The efficacies used in the main text were not reported this way, but rather as a proportional reduction in likelihood of infection, independent of degree.

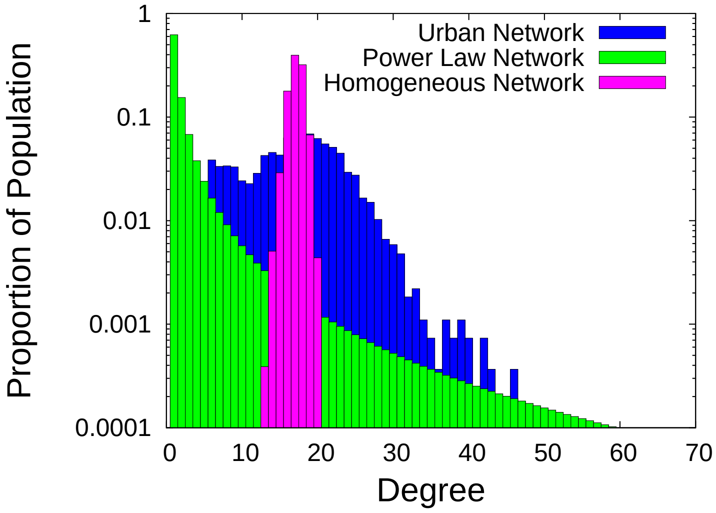

Figure S4: Log-linear plot of degree distributions for the Urban, Power Law, and Homogeneous networks. This corresponds to Figure 1B in the main text.

References

1. H. L. Smith. *Monotone dynamical systems: An introduction to the theory of competitive and cooperative systems.* American Mathematical Society, 1995.

2. Jean-Pierre Aubin. *Mathematical Methods of Game and Economic Theory.* Dover, 1979.
